# The impact of binder polarity on the properties of aqueously processed positive and negative electrodes for lithium-ion batteries

**DOI:** 10.1038/s41598-025-93813-9

**Published:** 2025-03-23

**Authors:** Andreas Weber, Noah Keim, Pirmin Koch, Marcus Müller, Werner Bauer, Helmut Ehrenberg

**Affiliations:** Karlsruhe Institute of Technology, Institute for Applied Materials, Karlsruhe, 76021 Germany

**Keywords:** Surface free energy, Aqueous processing, PVDF latex, CMC, Biopolymer, LiNi0.5Mn1.5O4, Chemistry, Energy science and technology, Materials science

## Abstract

The surface free energy of materials plays a crucial role in defining the interactions between interfaces. In this study, we introduce the theory behind surface free energy and extend its application to solvent-based manufacturing processes of positive (cathode) and negative (anode) electrodes for lithium-ion batteries. By employing binders, namely polyvinylidene difluoride latices and sodium carboxymethyl cellulose, with differing surface free energy compositions, we systematically investigate how surface free energy influences key electrode properties. The binder properties are shown to affect adhesion strength, electrical resistance, and water retention in electrodes, with analogous effects observed in both cathodes and anodes. For cathodes, these differences translate to measurable impacts on cell performance, particularly in terms of rate capability and long-term cycling stability. We also explore how binder induced variations in water retention influence the formation and stability of the solid electrolyte interphase. The findings highlight the critical role of the binder’s surface free energy composition in optimizing electrode manufacturing and provide new insights into the interplay between electrode surface chemistry, microstructure, and electrochemical performance.

## Introduction

Interactions of various interfaces dominate the properties of lithium-ion battery (LIB) electrodes. This expresses itself in various results regarding the resistance, mechanical strength, and translates into the cell performance. The microstructure of an electrode is dictated by the interplay among its constituent materials: active material, conductive additives, current collector, and binders. These components form complex networks of interfaces that govern electron transport, ionic conductivity, and adhesion. As the demand for high-performance batteries continues to rise, understanding these interfaces is critical to further optimize cells. The binder, while often considered a minor component in electrode formulations, plays a significant role in determining the electrode’s mechanical integrity, interfacial adhesion, and porosity. Binders must account for mechanical and electrochemical stability, good electrolyte wetting, while simultaneously accommodating the volumetric changes associated with electrochemical cycling. Additionally, binder properties directly influence the homogeneity of the various materials and, therefore, the quality of the final electrode. Given this multitude of interactions for binders it is highly necessary to further understand, how components influence the interactions of the binder with the inactive and active materials, e.g. $\hbox {LiNi}_{0.5}\hbox {Mn}_{1.5}\hbox {O}_4$ (LNMO), $\hbox {LiNi}_{0.8}\hbox {Mn}_{0.1}\hbox {Co}_{0.1}\hbox {O}_2$ (NMC811), $\hbox {LiFePO}_{4}$ (LFP), or graphite. Other industrial applications focusing on the creation of interfaces, such as painting and adhesive joining, have long established surface free energy (SFE) as a vital key figure.^1,2^ Thus far, in battery manufacturing the method remains underutilized. Ludwig et al.^3,4^ showcased the use of SFE to explain carbon-binder-domain and agglomerate formation in dry-processed cathodes. Li et al.^5^ demonstrated the influence of SFE on the wetting behavior of water-based slurries on aluminum current collectors. Their works relate to interactions in a gaseous atmosphere. However, slurry processing involves submerging different components in a solvent, either N-methyl-2-pyrrolidone (NMP) or water, rendering calculations regarding interactions between two surfaces in a gaseous atmosphere insufficient. By expanding of the theoretical framework, the application of SFE to interface formation during slurry processing is enabled. The main goal of this work is to bridge this gap to properly apply key figures resulting from the SFE. This includes introducing the theory required for applications with respect to interfaces in a gaseous and liquid environment. Additionally, we systematically investigate the influence of selected binders with regard to their surface free energy composition. The chosen binders are sodium carboxymethyl cellulose (CMC) and polyvinylidene difluoride (PVDF) in the form of a latex, which is a colloidal suspension of PVDF particles synthesized via emulsion polymerization in water and stabilized by surfactants. Finally, we use SFE to explain their impact on the electrode properties, water retention, and how these affect the resulting cell performance.

## Surface free energy and contact angles

**Fig. 1:**
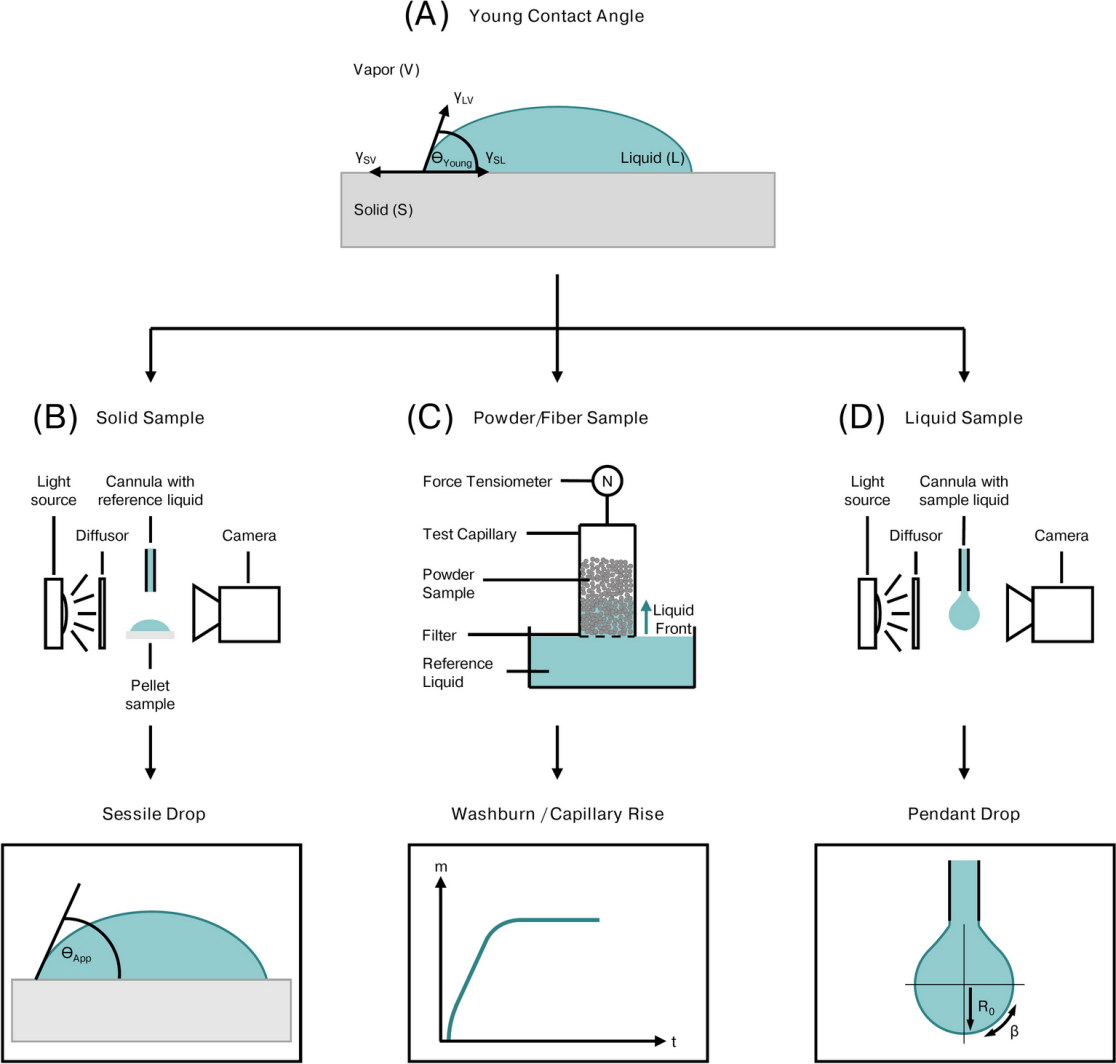
Flowchart of different contact angle measurement techniques depending on the investigated sample, based on the principle of the Young contact angle between a liquid droplet and a “perfect” solid surface.

The study of electrode properties is crucial for the design and optimization of efficient and effective electrochemical energy storage. One of the key factors that influence the performance of these devices is the interaction between all electrode materials. SFE, which is a measure of the energy required to create an interface between two surfaces, is a powerful tool for understanding the interaction between the electrode surface and its environment. The SFE of an electrode can be determined by measuring the contact angle of a liquid droplet on the surface. In the context of electrode development, the use of different binders can have a significant impact on the properties of the electrode. By applying the SFE methodology, key figures such as the free energy of interaction or the work of adhesion can be obtained, helping to better understand the underlying mechanisms responsible for various electrode properties. Figure 1 visualizes the process of the different types of contact angle measurements required for varying sample types.

### Theoretical background

When applying a liquid drop on the surface of a solid and non-swellable surface, a droplet forms.^6,7^ By adding a tangent to the profile of the resulting droplet at the three-phase interface of vapor (V), liquid (L) and solid (S), the corresponding contact angle $\theta$ can be determined, see Figure 1 (A).^6,8^ The value of the contact angle was first described by Young’s equation, showing the relation between the contributing interfacial tensions, resulting in an observable contact angle:^9^

1 $$\begin{aligned} \gamma _{SV} = \gamma _{SL} + \gamma _{LV} \cdot cos(\theta _{Young}) \end{aligned}$$

The Young equation is only viable for an ideal surface, which is smooth, non-deformable and shows no swelling behavior for the liquids.^6,10–12^ The apparent contact angle $\theta _{App}$ measured can vary from the contact angle described by the Young equation.^7^ This hysteresis is caused by the development of local energy minima, which result from a heterogeneous sample surface.^7,13,14^ Therefore, more advanced relationships were developed to better describe real samples. For instance, the Wenzel equation takes surface roughness and other properties of real samples into account.^8,10^ The Cassie-Baxter publication further expanded on the equation by considering air inclusion in between the surface and the droplet.^15^ Based on the Young equation, further work was done by Dupré.^16^ By expressing $\gamma _{SL}$ as a sum of facial tensions, the work of adhesion can be written as:

2 $$\begin{aligned} W _{SL}^{ad} = \gamma _{SV} + \gamma _{LV} - \gamma _{SL} = - \Delta G^{IF}_{SL} \end{aligned}$$

which is known as the Young-Dupré equation. The work of adhesion $W^{ad}_{SL}$ allows to calculate the theoretical force needed to separate the liquid from the solid. Inverse to $W^{ad}_{SL}$, the free energy of adhesion $\Delta G^{IF}_{SL}$ expresses the energy which is released during formation of an interface.^16–18^ This extension of the Young equation enables the study of $\gamma _{SL}$ through $W^{ad}_{SL}$, which can be directly determined by measuring $\theta _{App}$. While the work of adhesion $W ^{ad}_{SL}$ can be determined this way, to understand the surface tension and the contributing factors in more detail it required Fowkes to postulate the idea of partitioning the surface tension.^19–22^ Therefore $\gamma _{SV}$ has a number of contributors and is given by:

3 $$\begin{aligned} \gamma _{SV} = \gamma _{SV}^d + \gamma _{SV}^p + \gamma _{SV}^h + \gamma _{SV}^i + \gamma _{SV}^{AB} \end{aligned}$$

Here the contributions of the dispersion $\gamma _{SV}^d$, dipole-dipole interaction $\gamma _{SV}^p$, hydrogen bonding $\gamma _{SV}^h$, induced dipole-dipole interaction $\gamma _{SV}^i$, and acid-base components $\gamma _{SV}^{AB}$ for the surface tension $\gamma _{SV}$ are included. By considering a solid surface with only a dispersion component, Fowkes expressed the liquid-solid interfacial tension as:

4 $$\begin{aligned} \gamma _{SL} = \gamma _{SV} + \gamma _{LV} - 2 \sqrt{\gamma _{SV}^d \cdot \gamma _{LV}^d } \end{aligned}$$

which would allow the determination of $\gamma _{SV}$ trough $\theta _{App}$ after combination with the Young-Dupré equation, see Eq. (2). Nevertheless, it is only viable for materials with only disperse contributions to the surface. The Fowkes model was further expanded by Owens, Wendt, Rabel and Kälble (OWRK).^22–25^ By approximating that the solid surface tension and the liquid surface tension are composed of a dispersion component and a polar component, OWRK hypothesized that the polar interaction consists solely of hydrogen-bonding interactions. The resulting interfacial tensions are then expressed by:

5 $$\begin{aligned} & \gamma _{SV} = \gamma _{SV}^d + \gamma _{SV}^p \end{aligned}$$

6 $$\begin{aligned} & \gamma _{LV} = \gamma _{LV}^d + \gamma _{LV}^p \end{aligned}$$

Similar to the Fowkes model in Eq. (4), $\gamma _{SL}$ can be derived by assuming a geometric mean form of the polar components and is expressed by:

7 $$\begin{aligned} \gamma _{SL} = \gamma _{SV} + \gamma _{LV} - 2 \sqrt{\gamma _{SV}^d \cdot \gamma _{LV}^d }- 2 \sqrt{\gamma _{SV}^p \cdot \gamma _{LV}^p } \end{aligned}$$

Like Eq. (4), the expression can be combined with the Young-Dupré equation which leads to:

8 $$\begin{aligned} \gamma _{LV} (1+cos(\theta _{App})) = 2 \sqrt{\gamma _{SV}^d \cdot \gamma _{LV}^d }+ 2 \sqrt{\gamma _{SV}^p \cdot \gamma _{LV}^p } = - \Delta G^{IF}_{SL} \end{aligned}$$

with only two unknown terms $\gamma _{SV}^d$ and $\gamma _{SV}^p$ left. By determining $\theta _{App}$ for multiple liquids with known $\gamma _{LV}^d$ and $\gamma _{LV}^p$, the remaining terms can be calculated. The expression can be rewritten as:

9 $$\begin{aligned} \frac{\gamma _{LV} (1+cos(\theta _{App}))}{2\sqrt{\gamma _{LV}^d}} = \sqrt{\gamma _{SV}^p} \cdot \frac{\sqrt{\gamma _{LV}^p}}{\sqrt{\gamma _{LV}^d}}+\sqrt{\gamma _{SV}^d} \end{aligned}$$

To determine $\gamma _{SV}^d$ and $\gamma _{SV}^p$ a linear fit for multiple solvents is performed and then evaluated when $\gamma _{LV} (1+cos(\theta _{App}))/2\sqrt{\gamma _{LV}^d}$ is plotted against $\sqrt{\gamma _{LV}^p}/\sqrt{\gamma _{LV}^d}$.^26^ This process is schematically illustrated in Figure S1. All the aforementioned expressions are solely focused on a liquid system contacting a solid in a gaseous atmosphere. Ludwig et al.^3,4^ demonstrated that these conditions apply in dry powder mixing for dry electrode manufacturing, as the different electrode constituents are in direct contact with each other. However, they are not applicable when investigating a system submerged in water. Therefore, van Oss et al.^27^ made several differentiations of $-\Delta G^{IF}_{SL}$ to also be viable when submerged in a liquid.^27^

1. Work of adhesion between different particles of material 1, immersed in liquid 3
  10 $$\begin{aligned} \Delta G ^d _{131} = -2 (\sqrt{\gamma ^d_1}-\sqrt{\gamma ^d_3})^2 \end{aligned}$$
2. Work of adhesion between different materials 1 and 2, immersed in liquid 3
  11 $$\begin{aligned} \Delta G ^d _{132} = -2 (\sqrt{\gamma ^d_1}-\sqrt{\gamma ^d_3})(\sqrt{\gamma ^d_2}-\sqrt{\gamma ^d_3}) \end{aligned}$$

Eqs. (10) and (11) are equally valid with superscript p. Since the polar and dispersive component are additive, the overall free energy of adhesion is then given by:

12 $$\begin{aligned} \Delta G ^{IF} = \Delta G ^{d} + \Delta G ^{p} \end{aligned}$$

### Methodology

#### Sessile drop

When measuring a sample that provides a solid surface capable of holding a drop and does not interact adding a small volume of liquid on top of a dense surface, a droplet is forming. Using optical drop shape analysis (DSA) software, it is possible to detect the apparent contact angle, see Figure 1 (B). DSA creates a greyscale image of the droplet and automatically detects its outline. The contact angle is then determined by aligning a tangent with the outline.

#### Washburn / capillary rise

For samples that are not able to create a dense surface, the Washburn or capillary rise method has to be applied, see Figure 1 (C). The theory used to determine the properties of powders describes the rise of a liquid through a packed bed of powders or fibers. This is possible by approximating the flow through powders or fibers as equivalent to an array of tiny, parallel capillaries. By making this approximation, the Hagen-Poiseuille equation can be used to calculate the flow rate within these cylindrical capillaries:^28^

13 $$\begin{aligned} h^2(t)= \frac{r \gamma _L cos(\theta )_{adv}}{2 \eta } \cdot t \end{aligned}$$

*h* equals the height travelled by the liquid front, *r* is ascribed to the inner radius of the cylindrical capillary, $\gamma _L$ is the surface free energy of the reference liquid, $\eta$ equals the viscosity of the probe liquid, $\theta _{adv}$ is the advancing contact angle, and *t* is the time passed. Due to the difficulty of accurately determining the liquid front directly, using a force tensiometer is preferred. This method allows for the constant determination of the weight increase, indirectly determining *h* by following the mathematical relationship

14 $$\begin{aligned} m=\rho \phi V=\rho \phi \pi R^2h \end{aligned}$$

This allows for the direct correlation of the liquid height h and the mass gain m from the adsorbed liquid, by considering the liquid to from a cylindrical column with density $\rho$, radius *R*, height *h*, and the sample porosity $\phi$. This can be extended further to:

15 $$\begin{aligned} m^2(t)=r\phi ^2(\pi R^2)^2 \cdot \frac{\rho ^2\gamma _L cos\theta _{adv}}{2\eta } \cdot t \end{aligned}$$

The relationship is known as the modified Washburn equation, and is further simplified by defining the term of $r\phi ^2 (\pi R^2 )^2$ as the capillary constant *C*.^29,30^ The capillary constant is experimentally determined by using a fully wetting liquid with $\theta _{adv} = 0$. *C* is impacted by the particle size of the sample powder and the packing density in the sample holder, highlighting the necessity of consistent sample preparation. Uneven packing could result in an asymmetrical rise of the liquid front, tampering with the observed weight gain.^29,30^ The experimental setup of the Washburn method is schematically illustrated in Figure 1, with the encircled N depicting the force tensiometer and the following plot expressing the weight gain detected by the force tensiometer.

#### Pendant drop

When a liquid has an unknown surface free energy, it can be determined using the pendant drop method, illustrated in Figure 1 (D). In the first step, a small volume of liquid forms a hanging drop at the cannula of the measurement setup. Then an outline of the drop is automatically generated, leading to the determination of the size parameters $R_0$ and $\beta$. The liquid’s surface tension is then calculated via Eq. (16):

16 $$\begin{aligned} \gamma = \frac{\Delta \rho g R^2_0}{\beta } \end{aligned}$$

$\Delta \rho$ is depicting the difference in mass density, with *g* as the gravitational constant. As illustrated in Figure 1 (D), $R_0$ describes the radius of curvature at the apex of the drop, while $\beta$ is the shape factor. $\beta$ serves as control figure to indicate whether the fitting of the drop outline is sufficiently accurate. The most commonly applied model by Rotenberg et al.^31^ is based on a derivation of the Young-Laplace equation. To further distinguish between the polar and disperse contribution to the overall SFE, a contact angle measurement with a solid showing only disperse contributions, e.g. PTFE, is necessary.

## Results

### Cathodes

#### Surface free energy

Table 1 compiles the overall SFE, the dispersive, and the polar component of the tested electrode materials, designated as $\gamma _S$, $\gamma _S^d$, and $\gamma _S^p$, respectively. As shown in our previous publication, the aluminum current collector (CC), C65 and CMC have significantly larger dispersive than polar components while LNMO is an exception with a polar contribution of 30.0 $\hbox {mJ/m}^{2}$, a dispersive component of 25.6 $\hbox {mJ/m}^{2}$ and, therefore, a surface polarity of 54.0 %.^32^ Latex 1, 2, and 3 exhibit highly similar dispersive components of approx. 36 $\hbox {mJ/m}^{2}$, while varying in the polar component from 4.5 $\hbox {mJ/m}^{2}$ for Latex 1 to 11.3 $\hbox {mJ/m}^{2}$ for Latex 3. Latex 4, on the other hand, exhibits a significantly larger polar component of 31.6 $\hbox {mJ/m}^{2}$ and a smaller dispersive contribution of 22.4 $\hbox {mJ/m}^{2}$. When calculating the relative polarity of the latices this translates to Latex 1 having a surface polarity of 11.1 %, Latex 2 with 19.9 %, Latex 3 with 24.0 % and Latex 4 exhibiting the highest relative polarity of 58.5 %. By dividing the polar contribution $\gamma _S^p$ through the overall SFE $\gamma _S$ the polarity of the substrate is determined.

**Table 1:** SFE of different cathode materials calculated from contact angles with diiodomethane (DIM), dimethyl sulfoxide (DMSO), ehtylene glycol (EG), and water according to the OWRK method.

Solid sample	$\gamma _{S}$	$\gamma _{S}^d$	$\gamma _{S}^P$	Polarity
	$(mJ/m^2)$			($\%$)
Aluminum $\hbox {CC}^{[1]}$	35.5 ± 1.5	32.7 ± 1.3	2.8 ± 0.2	7.9
$\hbox {C65}^{[1]}$	31.7 ± 1.2	28.6 ± 1.0	3.1 ± 0.2	9.8
$\hbox {CMC}^{[1]}$	47.3 ±1.5	38.5 ± 1.2	8.8 ± 0.4	18.6
$\hbox {LNMO}^{[1]}$	55.6 ± 1.1	25.6 ± 0.9	30.0 ± 0.2	54.0
Latex 1	40.4 ± 0.5	35.9 ± 0.4	4.5 ± 0.1	11.1
Latex 2	43.7 ± 0.7	36.5 ± 0.6	7.2 ± 0.1	16.5
Latex 3	47.0 ± 2.8	35.7 ± 2.4	11.3 ± 0.4	24.0
Latex 4	54.0 ± 2.9	22.4 ± 2.4	31.6 ± 0.5	58.5

$^{[1]}$ Values reproduced from Weber et al.^32^.

#### Electrode Properties

**Fig. 2:**
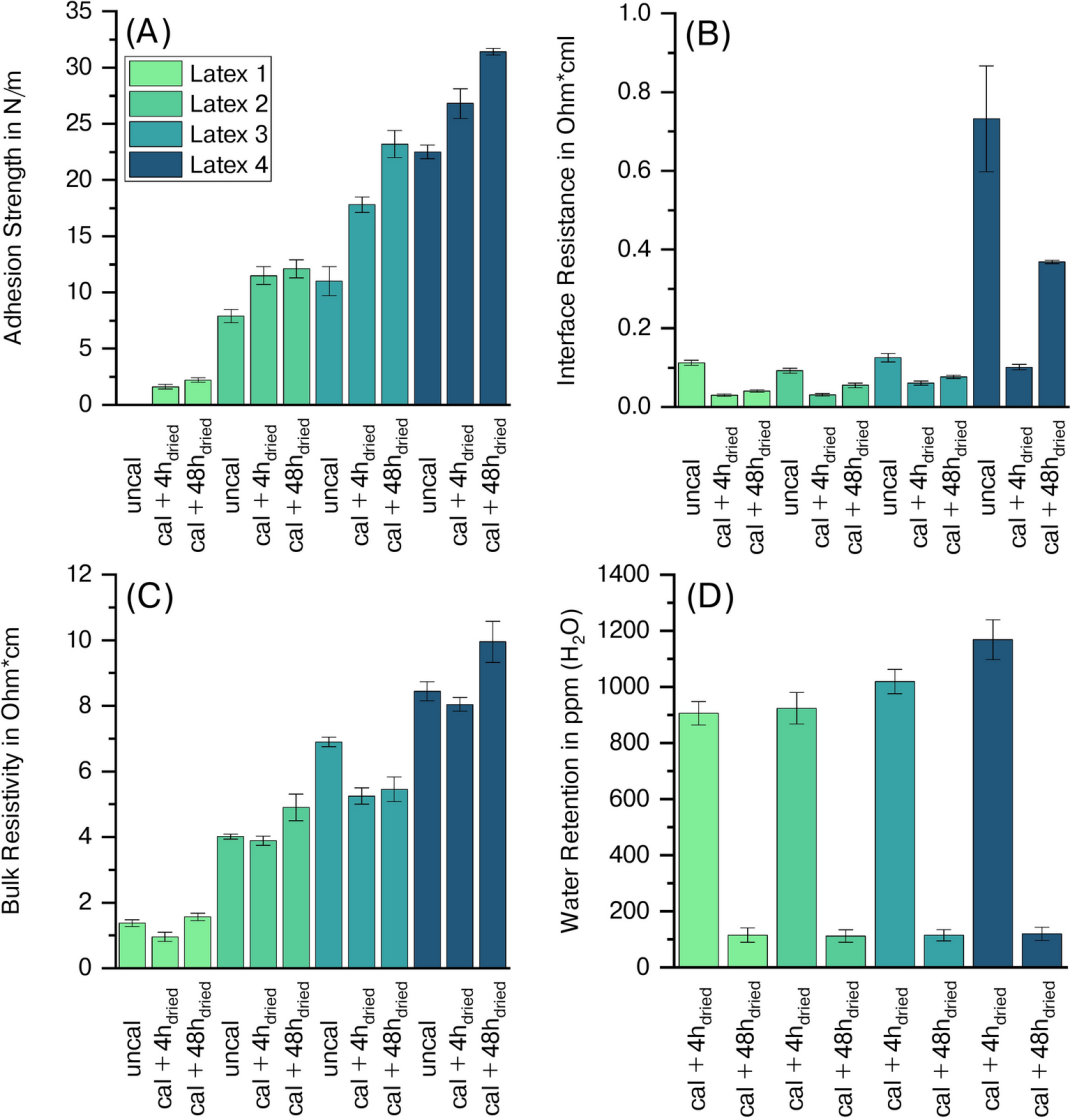
Adhesion strength (**A**), interface resistance (**B**), bulk resistivity (**C**), and water retention (**D**) of aqueously processed cathodes containing different PVDF latices.

The adhesion strength of the electrodes obtained from a 90 $^\circ$ peel test is illustrated in Figure 2 (A). Depending on the applied latex, the adhesion of the different cathodes varies significantly. Concerning Latex 1, it was not possible to prepare uncalendered samples for the adhesion strength measurement, as the cathode fully delaminated from the aluminum current collector at the slightest bending. Calendering and subsequent heat treatment, identical to secondary drying conditions before cell assembly (4 h or 24 h at 110 $^\circ$C under vacuum), led to a marginal improvement in adhesion strength from 0 N/m to 1.6 N/m and 2.2 N/m. In contrast, Latex 4 delivered a drastically higher uncalendered adhesion strength of 22.5 N/m, which increased further with successive heat treatment to 26.8 N/m after 4 h at 110 $^\circ$C under vacuum and 31.4 N/m after 24 h at 110 $^\circ$C and 24 h at room temperature, both under vacuum, resulting in 48 h.

Both interface resistance and bulk resistivity, depicted in Figure 2 (B) and (C), showed a strong correlation with the adhesion strength of the electrodes, with higher adhesion strengths being reflected in increased electrical resistance. Interface resistance increased with the polarity of the applied latex binder, from 0.1 $\Omega cm^2$ for Latex 1 to 0.7 $\Omega cm^2$ for Latex 4. Similarly, bulk resistivity increased from 1.4 $\Omega cm$ to 8.4 $\Omega cm$ for Latex 1 and Latex 4, respectively. It can further be observed that calendering of the cathodes initially decreases both interface resistance as well as bulk resistivity. However, during the subsequent drying step of 48 h, both values rise due to the partial melting of the insulating, thermoplastic PVDF, which is in line with previous findings.^32^

The cathodes were further investigated by Karl-Fischer-Titration, as visualized in Figure 2 (D). The residual water content of the cathodes after secondary vacuum drying for 4 h at 110 $^\circ$C ranged from 906 ppm up to 1168 ppm with Latex 1 containing the smallest amount of water which steadily increased towards Latex 4. By extension of the drying protocol to 24 h at 110 $^\circ$C and 24 h at room temperature, both under vacuum, resulting in overall 48 h, the residual water contents decreased significantly. The concentration of water retained in the cathodes then ranged from 110 ppm to 120 ppm, showing no difference between the distinctive latices. As a reference, an NMP-based cathode containing 4.6 wt% PVDF was also tested, which only retained around 140 ppm after vacuum drying for 4 h at 110 $^\circ$C. Following the extended drying protocol, the residual water content remained unchanged at 137 ppm.

#### Electrochemical characterization

**Fig. 3:**
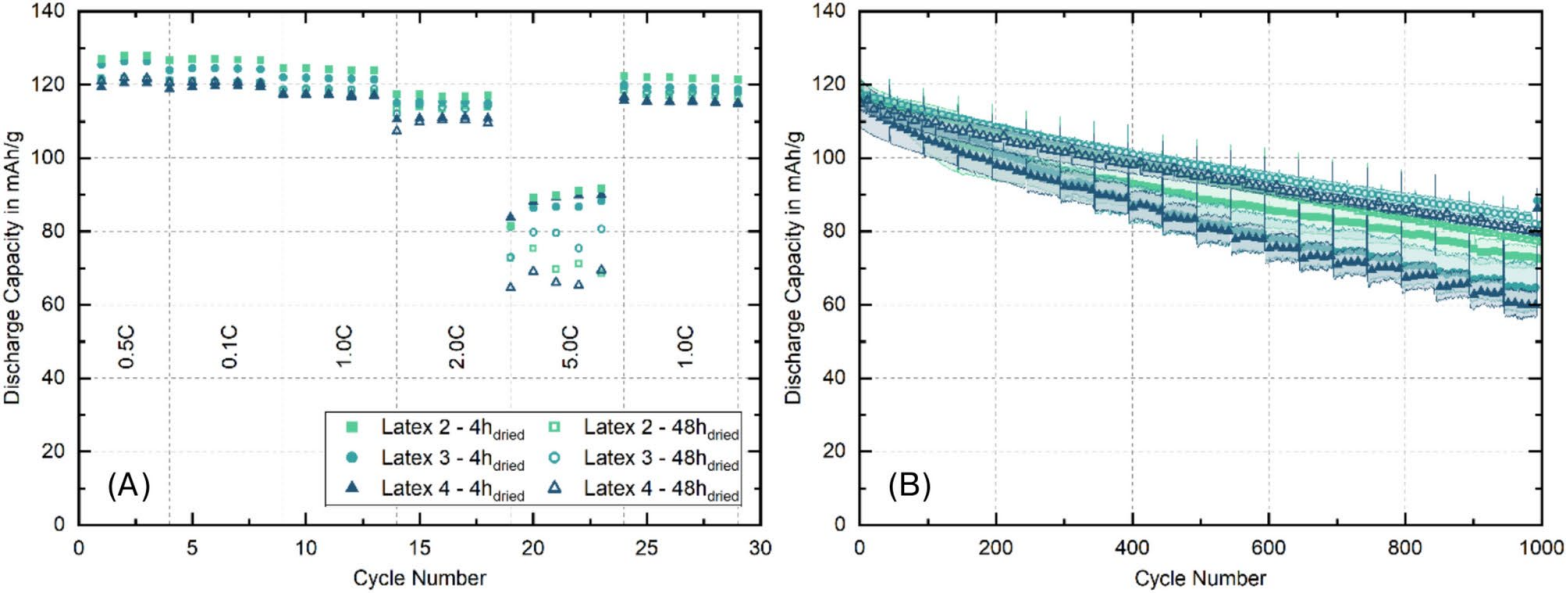
Comparison of rate capability (**A**) and long-term capacity retention (**B**) of cathodes containing different PVDF latices after 4 h and 48 h secondary drying.

Figure 3 illustrates rate capability (A) and long-term capacity retention (B) of the different cathodes after regular and extended secondary drying. Due to the very low mechanical integrity of Latex 1, electrode preparation is not possible as the electrode continually peels from the current collector during manual cell assembly. C-rate capability of the cathodes correlates with their overall electrical resistance, as illustrated in Figure 2 (B) and (C). The C-Rate test evaluates the specific discharge capacity (SDC) of the different electrodes containing the various latices at different discharge rates, while separately investigating the long-term behavior of the electrodes with the long-term investigations. At 0.1 C, all electrodes demonstrate a high discharge capacity, with Latex 2 (4h) reaching 127.6 mAh/g and Latex 3 (4h) at 126.0 mAh/g. However, drying for 48 hours consistently leads to slightly lower capacities, e.g., electrodes containing Latex 2 drop to 121.6 mAh/g and Latex 3 to 121.5 mAh/g. This trend continues at 0.5C and 1.0C, where 48h-dried samples generally show lower capacities than their respective 4h-dried equivalent. When increasing the C-rate the difference remains. For instance, at 2 C Latex 2 (4h) records 117.1 mAh/g, while the 48h dried electrodes are slightly lower at 113.8 mAh/g. Especially at 5 C the performance gap between drying times becomes more pronounced, with Latex 4 showing the largest difference in SDC which drops from 88.2 mAh/g (4h) to 66.9 mAh/g (48h). Despite showing higher resistance than Latex 2, Latex 3 shows slightly better rate performance at 5 C for the 48h dried counterpart. This can be summed up to the 4h dried electrodes performing better during the C-rate test, while the 48h dried electrodes lead to improved long-term stability. Latex 2, having the lowest resistance, delivers the highest average SDC of approx. 124.2 mAh/g, while Latex 4, having the highest electrical resistance amongst the investigated electrodes, reaches 117.3 mAh/g at a 1 C discharge rate. The tests further showed that the cathodes dried for 4 h outperformed the extensively dried cathodes at every C-rate. The performance ranking of the cathodes remains unchanged after extended drying, with the variations between the cathodes becoming more pronounced at 5 C. For a more detailed overview of the cell performance, the averaged SDC at the different C-rates as well as the SDC after 1000 cycles is given in tabular form in Table S1.

While Huttner et al.^33^ reported on the detrimental effects of extensive secondary drying on the capacity retention of cathodes due to embrittlement of the binding agent and the partial destruction of the percolation network, this work, applying CMC and PVDF latex in combination, found that extensive drying of 48 h leads to significantly less degradation for all tested cathodes. For cathodes dried for 4 h, capacity retention over 1000 cycles correlates with the residual water content, as determined by Karl-Fischer titration, see Figure 1 (D). Latex 2, retaining 924 ppm ($\hbox {H}_2$O), completes 1000 cycles with 61.2 % of the initial capacity. Latex 3 (1019 ppm ($\hbox {H}_2$O)) achieves 55.7 % and Latex 4 retaining the highest amount of 1168 ppm ($\hbox {H}_2$O) yields 52.6 % long term capacity retention. Following the extended drying procedure of 48 h, all cathodes demonstrate significantly improved degradation behavior, with Latex 2 retaining 65.9 % and Latex 3 and Latex 4 achieving even higher capacity retentions of approx. 69.5 %. Interestingly, the cathodes dried for 4 h display large standard deviations between the individual cells, indicating parasitic side reactions, which are drastically reduced after extended secondary drying. Thus, it can be stated that the degradation behavior of LNMO/graphite full cells is highly influenced by the amount of water retained in the cathode.

The anodes cycled against the cathodes containing the different PVDF latices were analyzed post-mortem by EDS, revealing distinctly pronounced SEIs, visualized in Figure S2 with the corresponding analysis data compiled in Table S2.

### Anodes

#### Surface free energy

Further, the anode components were investigated. The results for the total surface energies, including the separation into polar and disperse contributions by applying the OWRK method are summarized in Table 2.

**Table 2:** SFE of different anode materials, calculated from contact angles with DIM, DMSO and EG according to the OWRK method.

Solid sample	$\gamma _{S}$	$\gamma _{S}^d$	$\gamma _{S}^P$	Polarity
	$(mJ/m^2)$			($\%$)
Graphite	48.1 ± 1.8	45.7 ± 1.4	2.4 ± 0.4	5.0
$\hbox {C65}^{[1]}$	31.7 ± 1.2	28.6 ± 1.0	3.1 ± 0.2	9.8
SBR	40.6 ± 0.7	39.2 ± 0.5	1.4 ± 0.2	3.5
Copper CC	43.4 ± 0.8	42.2 ± 0.6	1.2 ± 0.2	2.8
$\hbox {CMC}_{0.5}$	49.0 ± 1.5	42.2 ± 1.2	7.0 ± 0.3	14.3
$\hbox {CMC}_{0.8}$	47.5 ± 1.0	36.9 ± 0.8	10.6 ± 0.2	22.4
$\hbox {CMC}_{1.0}$	46.7 ± 0.5	34.2 ± 0.4	12.5 ± 0.1	26.8
$\hbox {CMC}_{1.2}$	46.0 ± 1.8	32.2 ± 1.6	13.8 ± 0.2	30.1

$^{[1]}$ Value reproduced from Weber et al.^32^

The SBR (1.4 $\hbox {mJ/m}^{2}$) and copper CC (1.2 $\hbox {mJ/m}^{2}$) both show small polar contributions to the overall SFE, resulting in relative polarities of 3.4 % and 2.8 %, respectively. The graphite and carbon additive also exhibit significant differences in their polarity, despite both being carbon-based materials. Graphite has a lower polarity in comparison to C65. The active material shows a polar contribution of 2.4 $\hbox {mJ/m}^{2}$, while the C65 contribution is higher at 3.1 $\hbox {mJ/m}^{2}$. Due to the overall SFE of C65 being the lowest for all anode materials investigated, it translates to a high relative polarity of almost 10 %. Nevertheless, all anode constituents besides the CMC predominantly show high dispersive contributions to the overall SFE of the sample. For the CMC binder, the overall SFE increases for lower DS. In contrast, the polar contribution for $\hbox {CMC}_{0.5}$ is the lowest at only 7.0 $\hbox {mJ/m}^{2}$, equating to a relative polarity of 14.3 %. In comparison, an increasing number of substituents is accompanied by a higher polar contribution, which gradually increases from 10.6 $\hbox {mJ/m}^{2}$ for $\hbox {CMC}_{0.8}$, to 12.5 $\hbox {mJ/m}^{2}$ for $\hbox {CMC}_{1.0}$, and shows the highest polar contribution for $\hbox {CMC}_{1.2}$ at 13.8 $\hbox {mJ/m}^{2}$.

#### Electrode properties

**Fig. 4:**
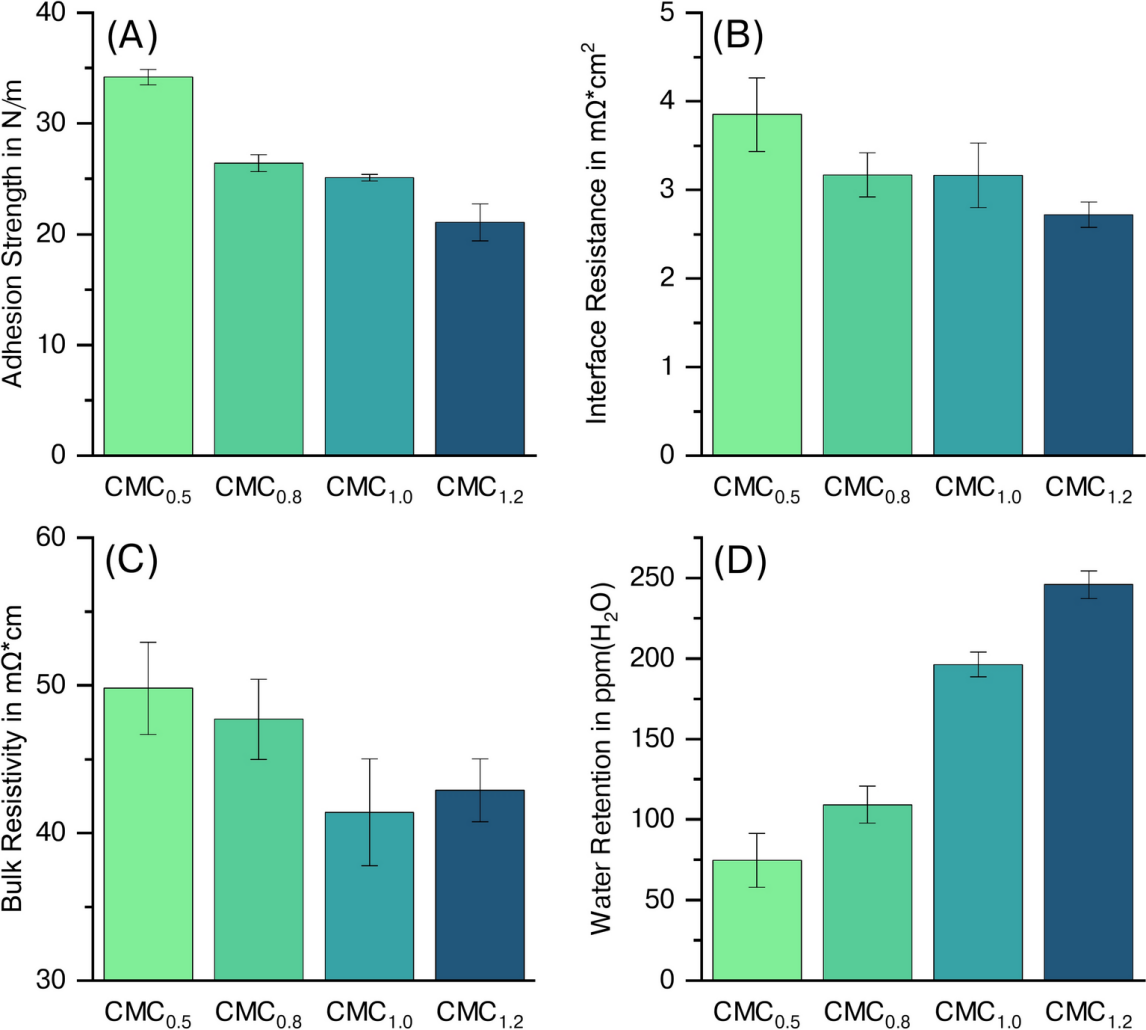
Adhesion strength (**A**), interface resistance (**B**), bulk resistivity (**C**), and water retention (**D**) of aqueously processed anodes containing CMCs with varying DS.

The adhesion strength of the anodes is visualized in Figure 4 (A). The results reveal a clear dependency of the electrode properties on the DS, which is in line with current findings. (Keim et al.) The lowest DS shows the highest adhesion strength, with $\hbox {CMC}_{0.5}$ at 34.2 N/m, followed by $\hbox {CMC}_{0.8}$ at 26.4 N/m and $\hbox {CMC}_{1.0}$ at 25.1 N/m, while $\hbox {CMC}_{1.2}$ exhibits the lowest adhesion strength at 21.1 N/m.

The results observed for the electrical resistance (see Figure 4 (B) and (C)) show that the electrodes containing higher DS CMCs lead to lower electrical resistance. For the interfacial resistance, anodes containing $\hbox {CMC}_{1.2}$ show the lowest results at 2.7 $m\Omega cm^2$. Decreasing the DS leads to a steady increase in interfacial resistance, with $\hbox {CMC}_{0.5}$ having the highest value at 3.9 $m\Omega cm^2$. A similar trend is observed for the bulk resistivity, where $\hbox {CMC}_{0.5}$ has the highest value at 49.8 $m\Omega cm$. The higher DS values all show a decrease in bulk resistivity, with $\hbox {CMC}_{0.8}$ at 47.7 $m\Omega cm$, $\hbox {CMC}_{1.0}$ at 41.4 $m\Omega cm$ and $\hbox {CMC}_{1.2}$ at 42.9 $m\Omega cm$.

Finally, the water retention of the individual dried anodes containing varying CMCs was measured and is summarized in Figure 4 (D). The results also show a dependency of water retention on the different CMCs. Dried anodes containing CMC with the lowest DS, which is $\hbox {CMC}_{0.5}$, had 75 ppm $(H_2O)$ of residual water, the lowest content compared to the other CMCs. An increase in the number of substituents, thereby increasing the DS, was accompanied by an observable increase in water, detectable via Karl-Fischer titration. For $\hbox {CMC}_{0.8}$ 110 ppm $(H_2O)$ were observed, further increasing to 200 ppm $(H_2O)$ for $\hbox {CMC}_{1.0}$. The electrodes containing $\hbox {CMC}_{1.2}$, which has the highest DS, also showed the highest water residue at 250 ppm $(H_2O)$.

## Discussion

### Cathodes

Eqs. (11) and (12) enable the calculation of $\Delta G_{132}^{IF}$ between the varying PVDF latices and the individual electrode components, as listed in Table 3, while submerged in water, based on the obtained SFE values.

**Table 3:** Free energy of adhesion of slurry components with different PVDF latices ($\Delta G^{IF}_{132}$ is annotated as $\Delta G^{IF}_{S-H2O-Latex}$ with 1 being the solid sample S, 3 the liquid ($H_2O$) and 2 the respective PVDF latex binder).

Solid sample	$\Delta G^{IF}_{S-H2O-Latex 1}$	$\Delta G^{IF}_{S-H2O-Latex 2}$	$\Delta G^{IF}_{S-H2O-Latex 3}$	$\Delta G^{IF}_{S-H2O-Latex 4}$
	$$(mJ/m^2)$$			
Aluminum CC	-57.8 ± 0.6	-51.7 ± 0.3	-44.2 ± 0.4	-16.8 ± 0.1
C65	-55.9 ± 0.5	-49.8 ± 0.4	-42.5 ± 0.6	-16.4 ± 0.4
CMC	-46.1 ± 0.5	-41.5 ± 0.3	-35.7 ± 0.1	-13.5 ± 0.8
LNMO	-17.7 ± 0.1	-15.9 ± 0.1	-13.7 ± 0.1	-5.1 ± 0.1

**Fig. 5:**
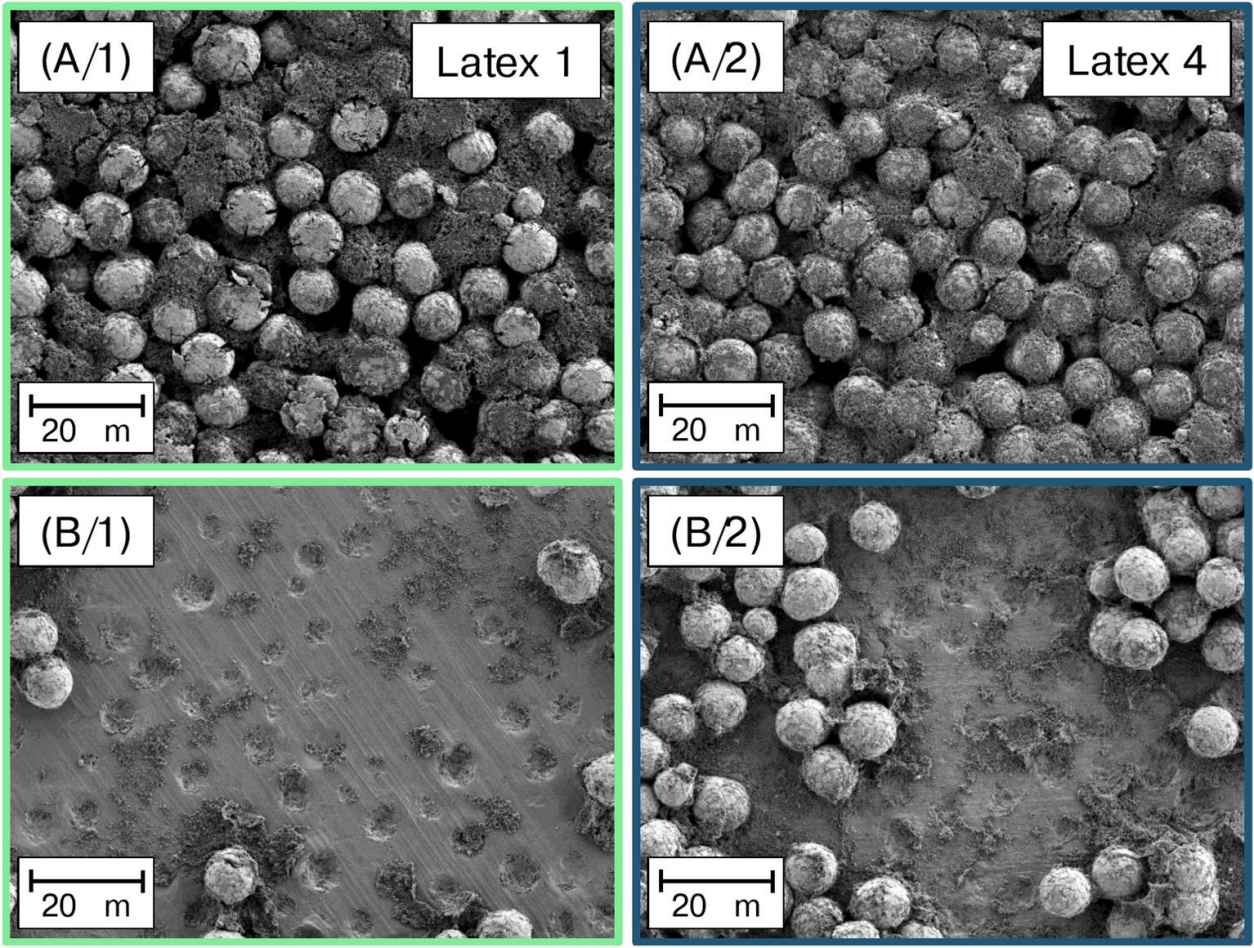
Comparison of SEM micrographs. A/1 shows the lack of interaction between dispersive Latex 1 and LNMO, resulting in pronounced CBDs while A/2 illustrates the homogeneous coverage of LNMO by CBDs when applying polar Latex 4. B/1 and B/2 show a blank aluminum current collector and an aluminum current collector with binder accumulated at its surface depending on the polarity of the PVDF latex, respectively.

All latices show the highest affinity towards the aluminum CC, as indicated by the largest amount of energy released during interface formation. C65 is the second most preferred surface of the latices, followed by CMC and lastly LNMO. The preferential order of adhesion while submerged in water can, therefore, be derived as CC>C65>CMC>LNMO and remains unchanged, regardless of the latex in use. During interpretation of the values, it must be kept in mind that all components, except the CC, undergo thorough dispersion during slurry preparation. Thus, $\Delta G^{IF}_{S-H2O-Latex}$ of the binders and the CC can initially be disregarded. When comparing $\Delta G^{IF}_{S-H2O-Latex 1}$ and $\Delta G^{IF}_{S-H2O-Latex 4}$, it can be observed that the difference in the free energy of adhesion between C65 and LNMO significantly decreases from 38.2 $\hbox {mJ/m}^{2}$ for Latex 1, to 11.3 $\hbox {mJ/m}^{2}$ for Latex 4. This suggests that when applying Latex 1, the binder has a much higher tendency to interact with C65 than with LNMO, while Latex 4 will also prefer the surface of C65, but to a much lesser extent, leading to a generally more homogeneous distribution of the latex throughout the electrode. The formation of carbon-binder-domains becomes less pronounced, and an increasing amount of free insulating polymer binder particles will become available with lessened interaction with C65. Similar to the findings of Barreras-Uruchurtu et al.^34^ on binder distribution in dry-coated electrodes, the more homogeneous distribution of PVDF will result in an increased bulk resistivity of the electrode, as evidenced in Figure 2 (C).^32,35–37^ Concerning the increased interface resistance, unbound polymer binder will tend to accumulate at the aluminum CC since the interface formation is most favored. The described differences in binder distribution were also evidenced by SEM, compiled in Figure 5. Top view SEM images of the cathodes show that Latex 1 interacts strongly with the CB while not interacting with LNMO. In contrast, applying Latex 4 leads to a homogeneous coverage of LNMO with carbon and binder. Additionally it can be observed that the aluminum CC is almost bare when using Latex 1 in comparison to Latex 4 which shows large amounts of residual latex accumulated at the surface of the aluminum after preparation of the sample via 90 degree peel off.

### Anodes

By applying Eqs. (11) and (12) to the aforementioned SFE results, depicted in Table 2, it is possible to calculate the free energy of adhesion for every slurry component with each separate CMC, assuming they interact while submerged in water, see Table 4.

**Table 4:** Free energy of adhesion of slurry components with different CMCs ($\Delta G^{IF}_{132}$ is annotated as $\Delta G^{IF}_{S-H2O-CMC}$ with 1 being the solid sample S, 3 the liquid ($H_2O$) and 2 the respective CMC binder).

Solid sample	$\Delta G^{IF}_{S-H2O-CMC0.5}$	$\Delta G^{IF}_{S-H2O-CMC0.8}$	$\Delta G^{IF}_{S-H2O-CMC1.0}$	$\Delta G^{IF}_{S-H2O-CMC1.2}$
	$$(mJ/m^2)$$			
Copper CC	-58.6 ± 0.8	-51.9 ± 0.9	-47.7 ± 0.8	-44.9 ± 0.6
SBR	-57.0 ± 0.6	-50.8 ± 0.7	-46.7 ± 0.6	-44.0 ± 0.4
Graphite	-55.5 ± 0.7	-49.3 ± 0.8	-45.2 ± 0.8	-42.5 ± 0.5
C65	-47.8 ± 0.1	-43.7 ± 0.2	-40.4 ± 0.1	-38.2 ± 0.1

The calculated values of $G^{IF}_{S-H2O-CMC}$ indicate that all CMC binders show the same preference regarding their affinity towards the other anode constituents. When submerged in water, the highest affinity of all CMCs, independent of their DS, is towards the copper CC, followed by the SBR binder, and graphite. CMC shows the lowest affinity for the conductive additive C65. Similar to the PVDF latices, the preferential order of adhesion remains the same, the absolute difference in the free energy of adhesion varies greatly. For $\hbox {CMC}_{1.2}$, the difference in free energy of adhesion between C65 and graphite is 4.3 $\hbox {mJ/m}^{2}$. Comparing this difference for the other CMCs reveals that the absolute difference steadily increases for lower DS. For $\hbox {CMC}_{1.0}$, the difference is 4.8 $\hbox {mJ/m}^{2}$, further increasing to 5.6 $\hbox {mJ/m}^{2}$ for $\hbox {CMC}_{0.8}$, and reaching its highest difference for $\hbox {CMC}_{0.5}$ at 7.7 $\hbox {mJ/m}^{2}$. Calculations of $G^{IF}_{S-H2O-CMC}$ reveal that the differences in electrical resistance and adhesive properties between the different anodes are directly influenced by the varying interaction of CMC. Due to the overall difference in the free energy of adhesion of $\hbox {CMC}_{1.2}$ being the smallest in comparison to the other CMCs, the binder is distributed equally among the different materials, as no surface creation is clearly more favorable than the others. On the other hand, the SFE calculations for $\hbox {CMC}_{0.5}$ indicate, that it tends towards the surfaces of graphite, SBR, and copper in comparison to C65.

The increase in adhesion strength from $\hbox {CMC}_{1.2}$ to $\hbox {CMC}_{0.5}$ can be attributed to more free binder as the interface formation with C65 becomes less favorable. While Lim et al.^38^ have shown that less adsorbed CMC in the system leads to less migration of SBR, thereby increasing adhesion strength, investigations of SBR-free systems show the same tendency of adhesion strength, see Figure S3. As CMC acts as dispersant during slurry preparation, the relative decrease in affinity from $\hbox {CMC}_{1.2}$ to $\hbox {CMC}_{0.5}$ towards C65 leads to less pronounced dispersion of the CB. This is accompanied by an increase in both bulk resistivity and interface resistance.

### Water retention

Calculations of $G^{IF}_{Binder-H2O}$, which is the free energy of hydration, i.e. the energy released during the formation of a hydration layer, show that for both PVDF latex and CMC, $G^{IF}_{Binder-H2O}$ becomes increasingly negative with higher polarity, see Table S3. The results strongly suggest a direct correlation between the binder’s polarity and its water-retaining capability as the hydrophilicity increases.^27^ High polarity binders, when subjected to the same amount of energy input during secondary drying, retain higher amounts of residual water in the electrodes, as more energy is required to remove the hydration layer. This was observed with both PVDF latices and CMC. When introducing a drastic surplus of energy during secondary drying, as shown for the cathodes, water retention is independent of binder polarity.

### Electrochemical characterization

Concerning the rate capability of the cathodes, the highest SDC was achieved by Latex 2, as it had the lowest electrical resistance paired with the lowest water retention. Both Latex 3 and Latex 4 ranked lower in SDC, which correlated well with their electrical resistance and water retention, showing that higher resistance leads to worse C-rate capability. The even lower SDCs of the extensively dried cathodes were attributed to their heightened electrical resistance as a result of further wetting by the PVDF latices. This decrease in SDC is due to the increase in electrical resistivity of the electrodes with higher drying durations. A higher electrical resistivity of the electrodes results in a more pronounced overpotential during charging and discharging. This overpotential leads to an incomplete sequence of the redox reactions in the respective voltage window, thereby decreasing the SDC. This is especially apparent at higher C-rates. Only Latex 3 (48h) at a discharge rate of 5 C shows a higher SDC than Latex 2 (48h) despite a higher electrical resistance. Considering the mean deviation of the SDC, the difference between Latex 2 (48h) and Latex 3 (48h) was not significant enough to be deemed a better rate capability. The detrimental effects of residual water on the performance of electrodes in LIBs are well documented in literature.^39–41^ Several authors have shown that water leads to a variety of parasitic side reactions, namely the formation of HF and the consequential dissolution of transition metal ions, e.g. $\hbox {Mn}^{2+}$, which is soluble in the electrolyte and known to travel to the anode where it causes a pronounced formation of SEI under loss of $\hbox {Li}^+$ inventory, as evidenced by EDS analysis, see Table S2.

## Conclusion

In this work we showcased the methodology and application of SFE to positive and negative electrodes for LIBs. Literature applying SFE in the context of batteries has primarily focused on the investigation of interactions in a gaseous atmosphere. By extending the scope to dispersions, it was possible to investigate the interactions between electrode components during water-based electrode processing, how these translate to the properties of the final electrodes, and consequently their electrochemical performance. In both cathodes and anodes, a lower affinity of the binder towards the CB resulted in less pronounced dispersion and more free binder, which expressed itself in higher adhesion strengths and heightened electrical resistance. Moreover, increasing polarity of the binders correlated with residual water retention in both the cathodes and the anodes after secondary drying. Electrochemical tests of the cathodes showed that the higher electrical resistance translated to decreased rate capability, while higher water retention negatively impacted capacity retention during long-term cycling. A decreased residual water content significantly improved the degradation behavior of the cathodes as a result of less parasitic, lithium-immobilizing side reactions.

## Experimental

### Electrode manufacturing

#### Cathodes

Electrodes were prepared using a disc agitator (Dispermat CA60, VMA Getzmann, Germany) with a 50 mm toothed disk at a tip speed of 5 m/s. The slurries consisted of 92.6 wt% LNMO (Haldor Topsoe, Denmark), 2.8 wt% conductive additive (C-NERGY Super C65, Imerys Graphite & Carbon, Switzerland), 1.8 wt% CMC (Walocel CRT 2000 PA 07, DuPont, USA), 2.8 wt% PVDF latex, and deionized water. The latices were provided by Arkema (France) (Latex 1: Kynar, experimental, particle size $\hbox {D}_{50}$ =145 $\upmu$m, Latex 2: Kynar, experimental, $\hbox {D}_{50}$=148 $\upmu$m, Latex 3: Kynar Flex Latex 32, $\hbox {D}_{50}$=152 $\upmu$m, Latex 4: Kynar Flex LBG4330X, $\hbox {D}_{50}$=149 $\upmu$m). All latices investigated have the same PVDF backbone and differ in their distinctive functionalization, which must not be disclosed in this work. The solid content of the slurries was adjusted to 60 wt%. Coating of the slurries onto an 18 $\upmu$m thick aluminum current collector (Batte-Foil-0004W, Pi-Kem, UK) was conducted by doctor blade on a role-to-role coating line (KTFS, Mathis AG, Switzerland). The web speed was set to 0.2 m/min. The areal capacity of the electrodes was set to 1.95 ± 0.05 $\hbox {mAh/cm}^{2}$. Drying of the electrode took place in-line in two consecutive convection ovens at 25 $^\circ$C. Although numerous studies have reported on the corrosion of aluminum current collectors caused by the high pH of aqueous LNMO slurries, drying at low temperatures has been found to effectively allow for the coating of the electrode without a detectable onset of corrosion.^42,43^ The electrodes were subsequently calendered at 50 $^\circ$C (GKL200, Saueressig, Germany) to a porosity of approximately 30 %. The corresponding anodes for full cell testing were manufactured by disc agitation (Dispermat CA60, VMA Getzmann, Germany) and coated onto a 10 $\upmu$m-thick copper current collector with an areal loading of 2.2 $\hbox {mAh/cm}^{2}$. The anodes are comprised of 96 wt% graphite (SMG-A3, Hitachi, Japan),1.5 wt% CB (C-NERGY Super C65, Imerys Graphite & Carbon, Switzerland), 1.25 wt% CMC (Walocel CRT 2000 PA, DuPont, USA), and 1.25 wt% styrene-butadiene rubber (SBR) (TRD 2001, JSR Micro, Belgium). The porosity was subsequently set to 48 % via calendering at 50 $^\circ$C (GKL200, Saueressig, Germany).

#### Anodes

Unless stated otherwise, the following formulation was used for all anodes: 96 wt% of graphite (natural, MechanoCap 1P1, H.C. Carbon, Germany), 1.5 wt% of carbon black (CB, C-NERGY Super C65, Imerys Graphite & Carbon, Switzerland), 1.25 wt% of SBR binder (TRD 2001, JSR Micro, Belgium) and 1.25 wt% of CMC. A variety of CMCs with different degrees of substitution (DS) were provided by IFF Speciality Products (Germany). The CMCs selected were $\hbox {CMC}_{0.5}$ (DS of 0.5, experimental), $\hbox {CMC}_{0.8}$ (DS of 0.8, experimental), $\hbox {CMC}_{1.0}$ (DS of 1.0, experimental), and $\hbox {CMC}_{1.2}$ (DS of 1.2, experimental). The slurry was mixed using a disc agitator (Dispermat CA60, VMA Getzmann, Germany). The water content was adjusted to achieve a solid content of 56 wt%. Electrodes were produced via roll-to-roll coating (KTFS, Mathis AG, Switzerland) via doctor blade onto a 10 $\upmu$m copper current collector with a wet-film thickness of 75 $\upmu$m. The two separate drying chambers had temperatures of 25 $^\circ$C and 30 $^\circ$C, respectively, with a web speed of 0.2 m/min. The areal capacity was set to 2.0 $\hbox {mAh/cm}^2$ ± 0.1 $\hbox {mAh/cm}^2$. All coatings were calendared to a density of 1.1 $\hbox {g/cm}^3$ (GLK200, Saueressig, Germany).

### Cell assembly

All electrodes had a diameter of 12 mm after punching. Before building the cells, the various electrodes were subject to secondary drying of 4 hours at 110 $^\circ$C under vacuum. This drying step was extended to equal 24 hours at 110 $^\circ$C, followed by an additional drying step of 24 hours at room temperature. After the secondary drying process was completed, the electrodes were transferred in an argon-filled glovebox (200B, M.Braun Inertgas-Systeme GmbH, Germany). The cells used a glass-fiber separator (Whatman GF/C, Sigma-Aldrich, USA), and 200 $\upmu$L of LP30 (1:1, V:V, EC:DMC, 1 M LiPF6, Sigma-Aldrich, USA) as the electrolyte, with 1 wt% of lithium bis(oxalato)borate (LiBOB, Sigma-Aldrich, USA) and 1 wt% of tris(trimethylsilyl) phosphite (TMSP, Sigma-Aldrich, USA) as electrolyte additive. A negative to positive electrode capacity (N/P) ratio of 1.1 was used with the anode slightly overbalanced. For each investigated polymer latex, three coin cells were built and tested each, with the mean average shown.

### Electrochemical testing

The protocol for the electrochemical characterization of the electrodes was the same for all cells. Two different protocols were applied, namely a rate test (Table 5) and a long-term test (Table 6), as depicted in the respective table. The cells were stored at 20 $^\circ$C and cycled within a voltage range of 3.5 V - 4.9 V vs $\hbox {Li}^+$/Li, considering the theoretical capacity of LNMO of 147 mAh/g. After assembly the coin cells were rested for 48 hours prior to testing. Each cell was charged up to 4.9 V in constant current mode, followed by a constant voltage step with a cut-off limit of 0.05 C.

**Table 5:** Rate test cycling protocol.

Cycle number	Discharging C-Rate	Charging C-Rate
0	48 hour resting period	
1-3	0.1	0.1
4-8	0.5	0.5
9-13	1	0.5
14-18	2	0.5
19-23	5	0.5
24-29	1	0.5

**Table 6:** Long-term cycling protocol.

Cycle number	Discharging C-Rate	Charging C-Rate
0	48 hour resting period	
1-3	0.1	0.1
4-1002	1	0.5
except: 53, 103, 153, 203, 253, 303,		
353, 403, 453, 503, 553, 603, 653,		
703, 753, 803, 853, 903, 953, 1003	0.1	0.1

### Adhesion strength measurements

Adhesion strength was measured in a 90 $^\circ$ peel-off test. Three 17 mm x 60 mm samples were measured. Each sample was pressed onto a double-sided adhesive tape with a load of 0.3 MPa for 10 seconds to enhance comparability. The sample was then placed in a zwickiLine Z2.5/TN (ZwickRoell, Germany), which pulled the sample at 600 mm/min while measuring the force needed for separation through a 10 N load cell over a distance of 40 mm, discarding the data from the first and last 10 mm of the sample. The average force was then divided by the width of the sample according to ISO 813:2019, resulting in the adhesion strength in N/m.^44^

### Resistivity measurements

Bulk resistivity and interface resistance were simultaneously determined via DC 4-terminal measurement (RM2610 Electrode Resistance Measurement System, Hioki E.E. Corp., Japan) based on the finite volume method. Each electrode was measured in five randomly chosen locations.

### Contact angle measurements

Contact angles of each electrode component were determined. Depending on whether the sample was porous or solid, either the Washburn method (K100, Kruess Scientific, Germany) or the sessile drop method (DSA30, Kruess Scientific, Germany) was applied. The Washburn samples were prepared by placing a defined amount of sample powder, not exceeding ± 1% between individual measurements, into a capillary tube with a radius of 6 mm. The powder density was then set by tapping the samples 50 times to a defined height in the capillary, based on a scale on the capillary tube. The sample amount and height of the different powders are given in Table S4. All measurements were conducted at 20 $^\circ$C. Multiple solvents were used to allow for the separation of the surface free energy into polar and disperse contributions. The physicochemical properties of the reference liquids used are compiled in Table 7. Exemplary raw data of the WB measurement of C65 is given in Figure S4.

**Table 7:** Physicochemical properties of reference liquids at 20 $^\circ$C^45^.

Solid sample	$\rho$	$\eta$	$\gamma _L$	$\gamma _L^d$	$\gamma _L^p$
	*g*/*mL*	$mPa \cdot s$	$(mJ/m^2)$		
n-Hexane	0.661	0.326	18.4	18.4	0.0
Diiodomethane (DIM)	3.325	2.762	50.8	50.8	0.0
Dimethyl Sulfoxide (DMSO)	1.100	1.996	44.0	36.0	8.0
Ethylene Glycol (EG)	1.109	21.81	48.0	29.0	19.0
Water	0.998	1.000	72.8	21.8	51.0

### SEM and EDS

SEM micrographs were taken using a Gemini (Carl Zeiss, Germany) at an acceleration voltage of 2 kV. EDS analysis was conducted using an Ultim Extreme (Oxford Instruments, UK) at an acceleration voltage of 4 kV.

### Water determination in electrodes (Karl-Fischer)

Residual water was determined via the coulometric titration in a Karl-Fischer setup with an additional oven mounted in before the Karl-Fischer titration chamber. Electrodes were dried using the same protocol, as if they were to be used in a cell. The measurement was conducted in a dry room with a dew point of -60 $^\circ$C. A sample with the dimensions of 17 mm by 60 mm was inserted into the sample vial, which was then heated to 160 $^\circ$C for 12 minutes, while the evaporating water was continuously transferred into the titration chamber via an inert carrier gas.

## Supplementary Information

Supplementary Information.

## Data Availability

The cathode data that support the findings of this study are available from Arkema SA but restrictions apply to the availability of these data as they are goverened by a confidentiality agreement and so are not publicly available. Data are however available from Andreas Weber upon reasonable request and with permission of Gregory Schmidt of Arkema SA. The anode data that support the findings of this study are available from International Flavors & Fragrances Inc. but restrictions apply to the availability of these data as they are goverened by a confidentiality agreement and so are not publicly available. Data are however available from Noah Keim upon reasonable request and with permission of Roland Bayer of International Flavors & Fragrances Inc.
