# Supplementary material for: The impact of binder polarity on the properties of aqueously processed positive and negative electrodes for lithium-ion batteries
Source: Sci Rep. 2025 Mar 23;15:10024. doi: 10.1038/s41598-025-93813-9 (PMC11930974; doi:10.1038/s41598-025-93813-9)
Supplement: Supplementary file 1 — Supplementary Information. [file 41598_2025_93813_MOESM1_ESM.pdf]

Supplementary Information: The Impact of Binder Polarity on the Properties of Aqueously Processed Positive and Negative Electrodes for Lithium-Ion Batteries

Andreas Weber[1,*,+], Noah Keim[1,+], Marcus Müller[1], Pirmin Koch[1], Werner Bauer[1], and Helmut Ehrenberg[1]

[1]Karlsruhe Institute of Technology, Institute for Applied Materials, Karlsruhe, 76021, Germany
[*]andreas.weber@kit.edu, noah.keim@kit.edu
[+]these authors contributed equally to this work

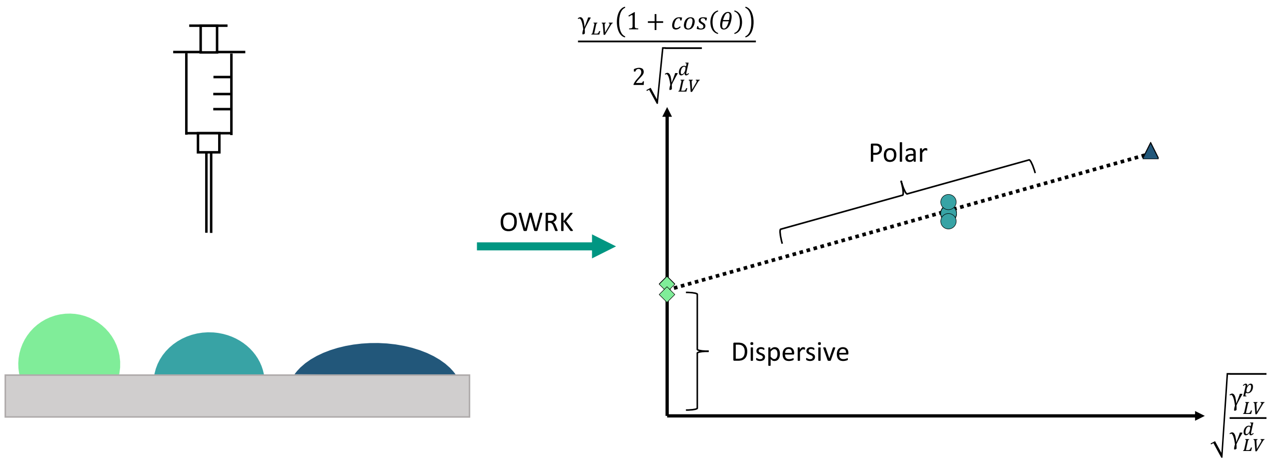

Figure S 1. Schematic illustration of the OWRK method to determine the polar and dispersive part, by using varying reference liquids, via geometric mean

Using the Owens-Wendt-Rabel-Kaelble (OWRK) method, an exemplary calculation is shown, which determines the surface free energy (SFE) of an C65 (carbon black) particle. By measuring the contact angles of polar and non-polar liquids, the dispersive and polar components of the SFE are derived. The reference liquids are water, dimethyl sulfoxide (DMSO), and diiodomethane (DIM). The corresponding x-value a measured contact angle is depicted for each reference liquid is determined by, with DMSO (γ_{LV}^p = 8 mN/m, γ_{LV}^d = 36 mN/m and being used as an example:

$$x = \sqrt{\frac{\gamma_{LV}^p}{\gamma_{LV}^d}} = \sqrt{\frac{8}{36}} \approx 0.47 \tag{1}$$

For DIM this results in a value of $x = 0$, DMSO $x \approx 0.47$, and water with $x \approx 1.57$. The respective contact angles of C65 with the reference liquids were 64.4 ° with DIM, 38.7 ° with DMSO, and 89.5 with water. The respective y-value is calculated by using the following equation, with DMSO being used as an example.

$$y = \frac{\gamma_{LV}(1+cos(\theta_{App}))}{2\sqrt{\gamma_{LV}^d}} = \frac{44\cdot(1+cos(38.7))}{2\cdot\sqrt{36}} = 6.53 \quad \sqrt{\frac{\text{mN}}{\text{m}}} \tag{2}$$

For DIM this results in a value of $y = 5.11$, DMSO with $y = 6.53$, and water with $y = 8.00$. By determining the geometric

mean of the resulting regression line, by using the LNMO as example, the y-intercept (5.35 $\sqrt{\frac{\text{mN}}{\text{m}}}$) and slope (1.76 $\sqrt{\frac{\text{mN}}{\text{m}}}$) are determined. Both are related to equation (9), which results in the y-intercept being equal to $\sqrt{\gamma_{SV}^{d}}$ and slope being equal to $\sqrt{\gamma_{LV}^{p}}$. This allows for the calculation of both γ_{SV}^{d} = 28.6 mN/m, and γ_{SV}^{p} = 3.10 mN/m.

After characterizing multiple solids, this allows for the calculation of the free energy of adhesion ΔG^{IF} between the different solids. When expanding the system to be investigated while submerged in a liquid, the free energy of adhesion ΔG_{132}^{IF} is determined. In this example, the interaction between C65 (1, γ_{SV}^{d} = 28.6 mN/m, γ_{SV}^{p} = 3.1 mN/m) and Latex 1 (2, γ_{SV}^{d} = 35.9 mN/m, γ_{SV}^{p} = 4.5 mN/m) is calculated, while the solids are submerged in water (3, γ_{SV}^{d} = 21.8 mN/m, γ_{SV}^{p} = 51.0 mN/m). The calculations are based on equations (11) and (12).

$$\Delta G_{132}^{d} = -2(\sqrt{\gamma_1^d} - \sqrt{\gamma_3^d})(\sqrt{\gamma_2^d} - \sqrt{\gamma_3^d}) \tag{3}$$

$$\Delta G_{132}^{d} = -2(\sqrt{28.6\frac{\text{mN}}{\text{m}}} - \sqrt{21.8\frac{\text{mN}}{\text{m}}}) \cdot (\sqrt{35.9\frac{\text{mN}}{\text{m}}} - \sqrt{21.8\frac{\text{mN}}{\text{m}}}) = -1.80 \ \frac{\text{mN}}{\text{m}} \tag{4}$$

$$\Delta G_{132}^{p} = -2(\sqrt{\gamma_1^p} - \sqrt{\gamma_3^p})(\sqrt{\gamma_2^p} - \sqrt{\gamma_3^p}) \tag{5}$$

$$\Delta G_{132}^{p} = -2(\sqrt{3.1\frac{\text{mN}}{\text{m}}} - \sqrt{51.0\frac{\text{mN}}{\text{m}}}) \cdot (\sqrt{4.5\frac{\text{mN}}{\text{m}}} - \sqrt{51.0\frac{\text{mN}}{\text{m}}}) = -54.1 \ \frac{\text{mN}}{\text{m}} \tag{6}$$

$$\Delta G^{IF} = \Delta G^{d} + \Delta G^{p} \tag{7}$$

$$\Delta G^{IF} = (-1.80 \ \frac{\text{mN}}{\text{m}}) + (-54.1 \ \frac{\text{mN}}{\text{m}}) = -55.9 \ \frac{\text{mN}}{\text{m}} \tag{8}$$

The result is equal to the one shown in Table 3, regarding the free energy of adhesion of slurry components with different PVDF latices and can be transferred to all the other calculations.

Discharge rate		cycles	Latex 2		Latex 3		Latex 4	
			$4h_{dried}$	$48h_{dried}$	$4h_{dried}$ (mAh/g)	$48h_{dried}$	$4h_{dried}$	$48h_{dried}$
Rate test	0.1 C	1-3	127.6 ± 0.3	121.6 ± 1.4	126.0 ± 0.3	121.5 ± 0.4	120.0 ± 3.5	121.5 ± 2.0
	0.5 C	4-8	126.8 ± 0.4	120.7 ± 0.3	124.2 ± 1.5	120.9 ± 0.1	119.4 ± 3.7	120.5 ± 0.1
	1.0 C	9-13	124.2 ± 0.7	118.3 ± 0.3	121.7 ± 2.2	118.7 ± 0.4	117.3 ± 4.1	117.1 ± 1.4
	2.0 C	14-18	117.1 ± 0.8	113.8 ± 0.7	115.2 ± 2.6	113.6 ± 0.6	110.9 ± 3.5	109.4 ± 1.1
	5.0 C	19-23	88.7 ± 2.6	71.5 ± 4.6	85.8 ± 4.0	77.7 ± 6.4	88.2 ± 1.0	66.9 ± 7.8
	1.0 C	24-29	121.9 ± 0.9	117.3 ± 0.5	119.1 ± 2.9	118.3 ± 0.4	115.3 ± 4.2	115.4 ± 1.6
Long-term	1.0 C	1000	72.6 ± 2.8	77.1 ± 0.3	64.7 ± 6.7	81.7 ± 1.5	60.4 ± 3.4	79.6 ± 1.2

Table S 1. Average specific discharge capacity of the different electrodes at varying discharge rates of the rate test and the last cycle of the long-term test

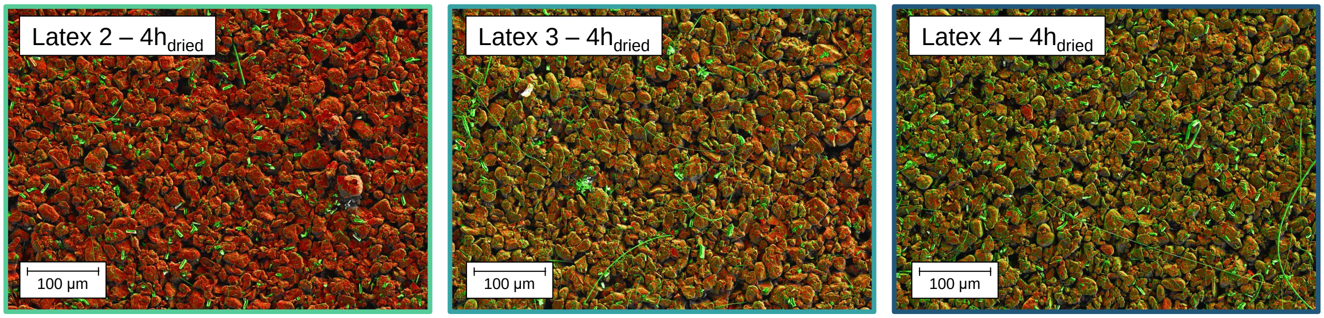

Figure S 2. Post-mortem analysis of anodes cycled against cathodes containing different PVDF latices. Layered electron images of carbon (red) and oxygen (green) show an increasingly more pronounced SEI formation

The exact elemental mappings are given in Table SI 2. Due to residual glass fibers (SiO_2), stemming from the separator, the oxygen content is adjusted by subtracting 2:1 O:Si atomic-%.

Element	Latex 2 - 4h$_{dried}$	Latex 3 - 4h$_{dried}$ (atomic-%)	Latex 4 - 4h$_{dried}$
C	76.1	69.1	59.1
O	15.1	21.1	26.2
B	3.5	3.4	3.5
F	2.7	2.7	4.3
P	1.6	1.7	2.5
Mn	0.2	0.3	0.6
Ni	0.0	0.0	0.0

Table S 2. Elemental mappings of anodes obtained from post-mortem cells after 1000 cycles against cathodes containing different PVDF latices analyzed by EDS.

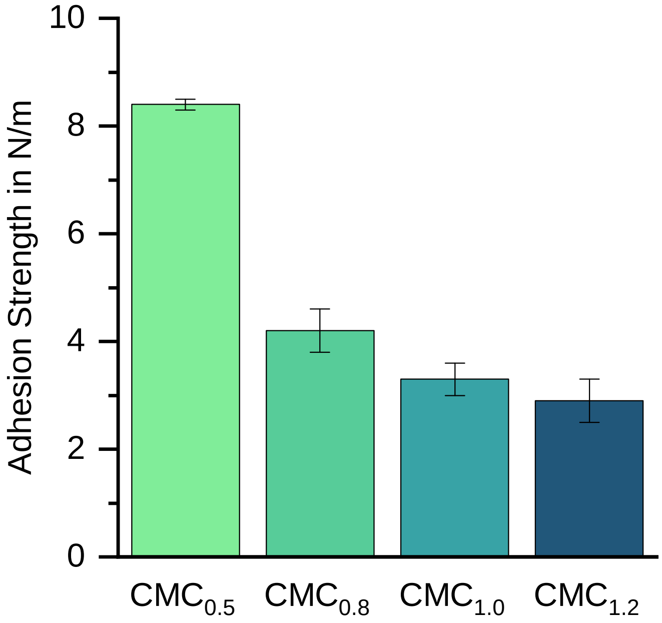

Figure S 3. Adhesion strength results for SBR-free anodes, with various CMCs. The SBR was substituted with graphite.

Solid sample	ΔG^{IF}_{S-H20} (mJ/m^2)
Latex 1	-86.1 ± 0.6
Latex 2	-94.8 ± 0.8
Latex 3	-103.8 ± 2.7
Latex 4	-124.5 ± 3.0
$CMC_{0.5}$	-98.3 ± 1.6
$CMC_{0.8}$	-103.3 ± 1.2
$CMC_{1.0}$	-105.1 ± 0.6
$CMC_{1.2}$	-106.1 ± 1.8

Table S 3. Calculation of ΔG^{IF}_{S-H20} for varying polymer binders.

Solid sample	Weight (mg)	Capillary Height (mm)
C65	100	15
LNMO	3000	15

Table S 4. Summary of the sample weight and power height in the capillary for samples measured via the Washburn method.

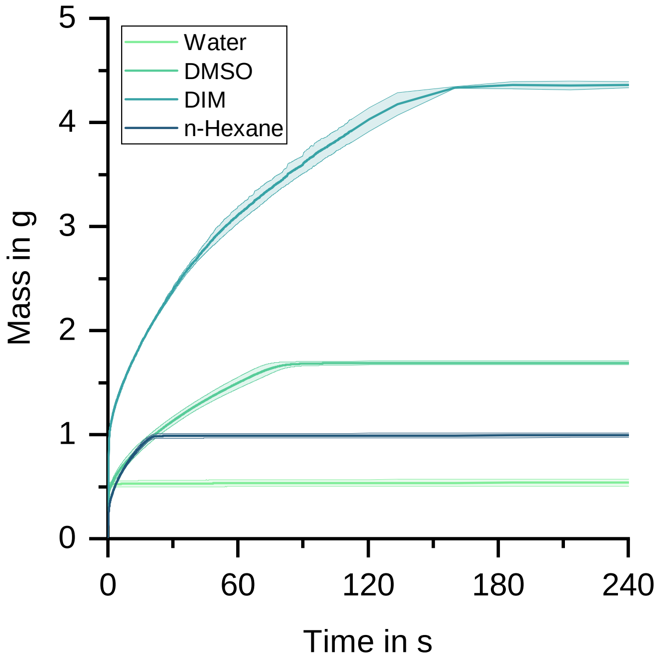

Figure S 4. Exemplary raw data of the WB measurement of C65 using n-hexane for the determination of the capillary constant as well as the reference liquids for the determination of the SFE. The data shown is the mean value of 3 individual measurements. The initial set-off in mass is due to the wetting of the filter on the bottom of the capillary.
